# The role of oral appliance therapy in obstructive sleep apnoea

**DOI:** 10.1183/16000617.0257-2022

**Published:** 2023-06-21

**Authors:** Ama Johal, Mona M. Hamoda, Fernanda R. Almeida, Marie Marklund, Harishri Tallamraju

**Affiliations:** 1 Oral Bioengineering, Institute of Dentistry, Queen Mary, University of London, London, UK; 2 Department of Oral Health Sciences, Faculty of Dentistry, University of British Columbia, Vancouver, BC, Canada; 3 Department of Otontology, Medical Faculty, Umea University, Umea, Sweden

## Abstract

There is now widespread recognition within the world of sleep medicine of the increasing importance of dental sleep medicine and, in particular, the role of oral appliance therapy (OAT) in the management of adults with obstructive sleep apnoea (OSA). For the purpose of this review, the term OAT refers to a custom-made intra-oral appliance, which acts to posture the mandible in a forward and downward direction, away from its natural resting position. Whilst nasally applied continuous positive airway pressure remains the “gold standard” in nonsurgical OSA management, OAT remains the recognised alternative treatment.

This review of OAT aims to provide an evidence-based update on our current understanding of their mode of action, exploring the potential anatomical and physiological impact of their use in preventing collapse of the upper airway; the current clinical practice guidelines, including the recently published National Institute of Clinical Excellence 2021 guidance, in conjunction with the American Academy of Sleep Medicine and American Academy of Dental Sleep Medicine; optimal design features, comparing the role of custom-made *versus* noncustom OAT devices and the importance of titration in achieving a dose-dependent effect; patient predictors, preference and adherence to OAT; its impact on a range of both patient- and clinician-centred health outcomes, with a comparison with CPAP; the limitations and side-effects of providing OAT; and, finally, a look at future considerations to help optimise the delivery and outcomes of OAT.

## Abstract 2

**A review for clinicians on the important role of oral appliance therapy in the management of adult patients with OSA. It addresses the key areas of mode of action, rationale, practice guidelines, predictors and their impact on a range of health outcomes.** https://bit.ly/3L63vmi

## Introduction

Obstructive sleep apnoea (OSA) is a common sleep-related breathing disorder, characterised by repeated collapse of the pharyngeal airway with resultant apnoeas, hypopnoeas and sleep arousal. Furthermore, there is recognition of the significant adverse health outcomes of untreated OSA for the patient, their partner and the wider community [1]. Severe long-term effects of this disease include excessive daytime sleepiness, cognitive dysfunction, hypertension, impaired quality of life and increased cardiovascular morbidity and mortality [2]. Thus, whilst continuous positive airway pressure (CPAP) remains the most efficacious and commonly prescribed treatment in OSA, the importance of understanding the role and use of oral appliance therapy (OAT) as an acceptable alternative should be a key part of our multidisciplinary care pathway. OAT is a noninvasive treatment for mild–moderate OSA and reduces apnoeas/hypopnoeas by enlarging the cross-sectional upper airway dimension *via* anterior displacement of the mandible and the attached tongue, resulting in improved upper airway patency [3, 4]. Furthermore, other nonsurgical treatments include lifestyle change and weight reduction, as well as behavioural modifications such as changing sleep postures. However, the former is advised for mild OSA nonsymptomatic OSA patients and the latter is prescribed for mild positional sleep apnoea [5].

## Terminology and classification

An overview of the nomenclature of oral appliances used to treat OSA is also worth considering, given there is no international agreement on their terminology. There remains a range of terms, from mandibular advancement splints/device/appliances, through to mandibular repositioning appliance/devices. All of which act in common to posture the mandible, to varying degrees, in a forward and downward direction, away from its natural resting position. Furthermore, oral appliances can be broadly classified into custom-made (titratable/nontitratable) and noncustom or “boil and bite” devices (titratable/nontitratable). The former are made directly from either a physical impression or digital scan of the patient's dentition and a bite registration. They are either manufactured as a single appliance, known as a monobloc, incorporating a pre-determined degree of mandibular protrusion or as a dual appliance, with separate components for the maxillary and mandibular dentition and some integral “locking” mechanism that subsequently permits adjustment in the amount of mandibular advancement required. The present review will use the term OAT to describe a custom-made titratable appliance.

In contrast, noncustomised appliances are purchased directly by the patient, in a pre-fabricated state and typically offer a limited, if any, scope for adjustment of mandibular protrusion. Some manufacturers have attempted to develop semi-customised devices, in which the patient themselves attempts to undertake a moulding of their maxillary and mandibular dentitions, prior to returning for fabrication of the appliance. The pre-fabricated “boil and bite” offer the advantages of being more readily available and less costly. However, a recent systematic review and meta-analysis demonstrated that not only customised OAT significantly more effective in treating OSA, but they were also much better tolerated and preferred by patients, due to their superior retention and fit [6]. As such, the custom-made titratable appliance remains the principle recommended choice of treatment (see below).

## Mode of action

The pharyngeal airway is rather unique in structure, being completely devoid of any skeletal framework, which in turn leaves it highly susceptible to collapse of its surrounding anatomical soft tissues. A range of imaging modalities have allowed us to better understand the impact of OAT on the pharyngeal airway space and therefore the role these devices play in alleviating OSA. In line with the multifactorial aetiology of collapse of the pharyngeal airway, OAT may well act by a variety of mechanisms, which can be classified as follows.

### Anatomical

#### Direct anatomical action

In posturing the mandible in a downward and forward direction, OAT directly acts to increase the size of the pharyngeal airway through a range of muscular attachments to the mandible (*e.g.* genioglossus) [7], as such the tongue is drawn forwards and to a lesser extent the soft palate through further muscular attachments (*e.g.* palatoglossus and pharyngeal constrictor muscles). Thus, we observe an increase in the post-lingual (*i.e.* oro- and hypo-pharynx) and post-palatal (*i.e.* velo-pharynx) airway spaces [8–12]. However, through the use of 3D videofluoroscopy, magnetic resonance imaging, computerised tomography and drug-induced sleep nasendoscopy (DISE), we have come to appreciate that the resultant increase in airway dimension is not limited to the antero–posterior dimension [13, 14]. In fact, the greatest observed increase is in the lateral dimension, thus accounting for the significant improvement observed in response to OAT [10]. Several imaging studies using cone-beam computer tomography, magnetic resonance imaging (MRI) or DISE have demonstrated a significant increase in upper airway volumes due to mandibular advancement, predominantly in the velopharynx region [15–17]. Furthermore, MRI investigating the effects of mandibular advancement on the tongue observed an increase in vertical tongue length in the anterior region [18]. The authors also observed an increase in the distance from the soft palate as the tongue moved caudally [18]. The authors attributed these changes to the thickness of the oral appliance worn and increase genioglossus activity due to mandibular protrusion [19–21]. In relation to the soft palate, the authors found a decrease in the area and width due to mandibular advancement [18]. These findings are consistent with studies using cephalometric analysis [22, 23].

#### Indirect anatomical action

If we consider for a moment the pharyngeal airway as analogous to a long thin collapsible tube. It is then conceivable that OAT in posturing the mandible forward places the surrounding soft tissues under tension and thereby acts indirectly to prevent pharyngeal airway collapse, much like stretching a long thin party balloon, by pulling at each end would ensure it did not collapse. This structural change may also account for the observed reduction in CPAP pressure and improved comfort when used in conjunction with OAT [24]. The pharyngeal/upper airway patency is balanced by factors such as the sub-atmospheric intraluminal pressure during inspiration and upper airway dilator muscle activity influenced by the upper airway dimensions and neuromuscular reflex interactions. Ng *et al*. [3] demonstrated that OAT significantly reduces the upper airway closing pressures in moderate sleep apnoea patients in different stages of sleep. The mechanism of action behind the improvement in upper airway collapsibility remains unclear; however, Isono *et al*. [25] attributed this to the stretching of the soft palate during mandibular protrusion. This leads to the tightening of the velopharynx because of the connection of the lateral wall of the soft palate to the base of the tongue through the palatoglossal arch. Similarly, Bamagoos *et al*. [26] demonstrated that improvement in upper collapsibility is significantly associated with different mandibular advancement positions *i.e.* 0% “habitual bite”, 50% and 100% of maximal comfortable mandibular advancement. The authors observed a significant reduction in the upper airway closing pressures in a dose-dependent manner across the three mandibular positions. However, no change was observed in the genioglossus muscle activity and responsiveness.

#### Physiological action

**FIGURE 1:**
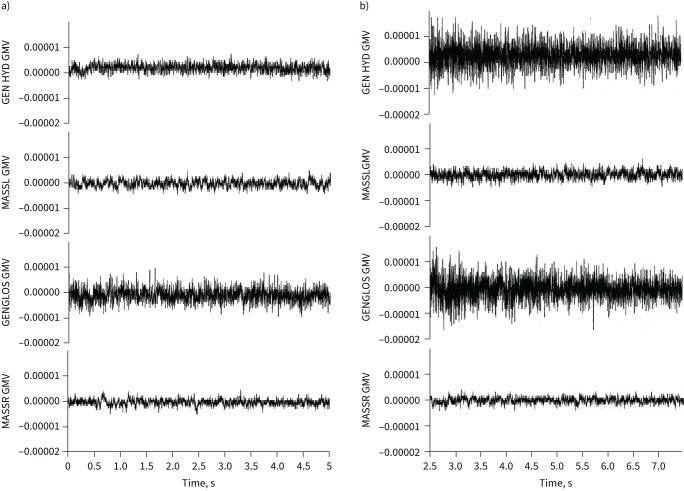
a) Electromyograph showing resting muscle activity in the right masseter (MASSR), left masseter (MASSL), genioglossus (GENGLOS) and geniohyoid (GEN HYD). b) Electromyograph showing increased muscle activity following oral appliance insertion in the genioglossus (GENGLOS) and geniohyoid (GEN HYD) dilator airway muscles. GMV: grams-microvolts.

Pharyngeal airway patency is maintained through the action of a range of upper airway dilator muscles. The role of OAT in stimulating upper airway dilator muscle action was explored using bipolar surface electrodes to assess genioglossus and geniohyoid muscle activity [20]. The researchers found that a significant stimulatory effect was observed in response to mandibular advancement and a potential physiological action proposed (see figure 1). This has been anecdotally reported by patients, who note reduced levels of symptoms over a period of proceeding nights, when they stop using their OAT, after continuous use. Equally, this “carry-over effect” has both been observed and is accounted for in a number of crossover clinical trials, necessitating a sufficiently adequate intervening washout period [27]. More recently, a number of research teams have explored the role of hypoglossal nerve stimulation, using direct and indirect stimulation, and discovered a potential role for genioglossus muscle stimulation in the management of OSA [28–30]. However, as mentioned above, Bamagoos *et al*. [26] failed to observe any changes in genioglossus activity with mandibular advancement and argued that pharyngeal muscle functions are less likely to lead to an improvement in OSA patients using OAT. Nonetheless, the findings should be interpreted with caution as the study was limited to a small sample size (n=18) with predominantly obese patients with severe OSA, rendering the findings less generalisable [26]. Conversely, Almeida *et al.* [19] detected a decrease in the genioglossus muscle activity in a case report of a 54-year-old male patient with severe OSA with mandibular protrusion and associated the therapeutic outcomes of OAT with anatomical changes instead of upper airway muscle activity. However, given the inherent limitations of a single case report, further studies are needed with a larger sample size to confirm the association between mandibular advancement and genioglossus muscle.

## Clinical guidelines and practice parameters for OAT

There are currently two key evidence-based clinical guidelines and practice parameters available for clinicians on the use of OAT in OSA. The American Academy of Sleep Medicine and the American Academy of Dental Sleep Medicine produced a joint updated guideline in 2015 [31]. The guideline recommended that OAT be prescribed for adult OSA patients who were intolerant of CPAP or, importantly, expressed a preference for OAT.

More recently, in the UK, the National Institute of Clinical Excellence [5] published guidance recognising the role of OAT in OSA. It too suggested that adult patients with OSA who are unable to tolerate or declined CPAP should be offered a customised or semi-customised OAT as an alternative. However, caution was to be exercised with semi-customised OAT and that they may be inappropriate, due to their potentially impaired fit, for those with:

- active periodontal disease or untreated dental decay;
- few or no teeth;
- generalised tonic-clonic seizures.

Both guidelines recognised the importance of OAT being provided by a trained dentist, familiar with dental sleep medicine to obtain informed consent, select the optimal design and titration level, oversee both short- and long-term follow-up, and ensure optimum comfort and therapeutic effectiveness. They also highlight the opportunity of considering a follow-up sleep study with the OAT *in situ* to objectively assess changes in sleep physiology.

## Oral appliance design features

With the emergence of a plethora of different designs, little evidence exists of the potential benefits of different OAT designs, as such the following provide clinical guidance on the principal characteristics that should be sought:

1. Retention. Oral appliance retention has been defined as “resistance of the appliance to vertical movement away from the tissues” [32] and as “that quality inherent in the oral appliance acting to resist the forces of dislodgement along the path of insertion” [33]. Optimal tooth retention is a prerequisite for any OAT to ensure maximum therapeutic benefit. If the appliance becomes disengaged from the dentition during sleep, it will naturally fail to maintain the mandible in its forward posture, but rather allow it to rotate downwards and backwards (see 4), below). Whilst there is no specific number of teeth required for OAT provision, the greater the number of healthy teeth in the jaw, the greater the retention. Millman *et al*. [34] suggested at least six teeth be present per jaw, with a distribution of one or more being posteriorly positioned. It should be noted that OAT has been provided to edentulous patients, with the use of dental implants but this may require pre-implant surgery to ensure sufficient bone to accommodate the implants [35].
2. Mandibular titration. Given that patients with OSA can present with a range of severity, it is not possible to predict the exact amount of mandibular protrusion necessary for any given patient. Contemporary designs of oral appliances should permit the mandible to be advanced in a gradual and incremental manner to achieve maximum therapeutic benefit [31, 36–38]. A range of different advancement mechanisms is available, dependent on the OAT design. This titration phase may last up to a few months in OSA patients and ideally can be undertaken by the patient (under instruction) if the OAT design incorporates a self-adjustment mechanism of advancement, which becomes very cost-effective. Alternatively, this can be performed by a trained dentist but, as such, requires regular review. Whilst the titration process has the short-term disadvantage of not providing immediately effective treatment, it offers the greater long-term advantage that the patient can acclimatise to the OAT, in the absence of discomfort in the teeth or jaws and thereby increasing the likelihood of improved patient adherence. Furthermore, applying the principle of personalised medicine, the importance of determining a dose-dependent effect specific to the patient's needs, minimises the risks of unwanted tooth movements as a side-effect of OAT. On this basis, current appliances can be classified into first-, second- or third-generation OAT (table 1). Whilst there is evidence from an experimental setting, in which a dose-dependent effect is achieved by mandibular titration using a remote advancement activation, this is yet unpractised in routine patient care with OAT [39]. Thus, clinicians routinely advise patients to use subjective criteria, such as symptomatic improvement in determining the amount of mandibular titration required to achieve maximum therapeutic effect. Furthermore, this is also a cost-effective approach to adopt, before organising a follow-up sleep study to determine the impact of OAT on sleep physiology.
3. Occlusal coverage. In providing OAT, it is important that trained dentists ensure that the device covers the occlusal surfaces of all teeth present. This ensures that no unwanted vertical change (overeruption) of posterior teeth takes place and, in turn, prevents any occlusal interferences and bite disturbances.
4. Minimal anterior vertical opening. The accompanying muscle relaxation, during sleep, typically permits the mandible to rotate downwards and backwards, with an accompanying reduction in tongue space and narrowing of the pharyngeal airway space. Whilst OAT aims to advance the mandible, it is therefore an important design feature that such devices also act to minimise the amount of vertical opening, in order to achieve their optimal effectiveness [12, 40].

**TABLE 1:** Classification of the oral appliances into first-, second- and third-generation designs

**Generation**	**Design**	**Limitations/advantages**
**First (** **figure 2** **)**	One piece, monobloc	• No opportunity for incremental advancement, as the appliance is fabricated with a pre-determined forward position. • Higher degree of discomfort and decreased patient adherence.
**Second (** **figure 3** **)**	Two-piece, duobloc	• Permits incremental advancement but requires clinician input. • Less cost-effective.
**Third (** **figure 4** **)**	Two-piece, duobloc (regarded as the “gold standard”)	• Permits the patient to self-adjust the amount of mandibular advancement in an incremental manner. • More cost-effective as requires minimal clinician input.

**FIGURE 2:**
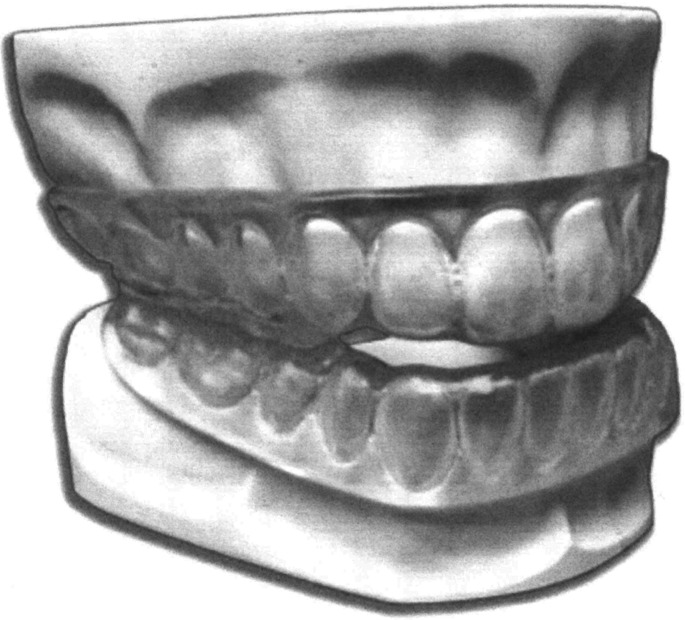
Monobloc (first generation).

**FIGURE 3:**
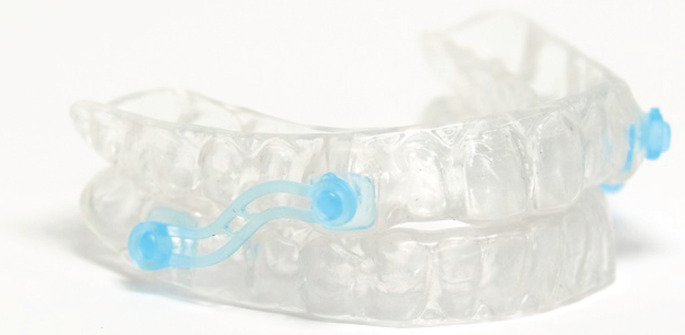
Example of a second-generation oral appliance therapy, in which there is some scope to adjust the amount of mandibular advancement. This requires the side connector (blue) bars to be removed and replaced by a shorter bar.

**FIGURE 4:**
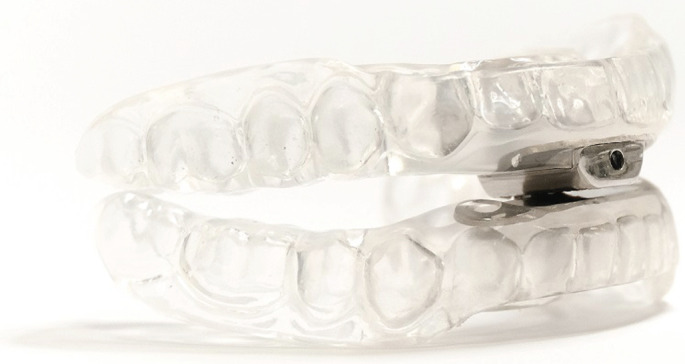
Example of a third-generation oral appliance therapy in which the patient can readily advance the mandible in increments of 0.25 mm with a simple adjustment allen key.

## Patient adherence and treatment responders

Patient adherence to OAT was limited to subjective data [41–43] until the introduction of objective monitoring of oral appliance wear time with the help of a thermal microsensor. The microsensor, embedded in the oral appliance design, calculates the actual wear time by measuring temperature every 15 min and then transforms this information into wear time when the temperature ranges between two specific values. In comparing subjective adherence data with objective data, Dieltjens *et al*. [44] reported a mean wear time of 7.2 h (6.2–7.8 h) and 7.1 h (6.7–7.7 h), respectively. Furthermore, a mean overestimation of 30 min is observed between subjective and objective adherence data [44, 45]. Based on patient-reported data, adherence to OAT appears to decline over time [46]. Hoffstein *et al*. [22] reported a wide range of adherence (4–76%) in the first year of appliance use. In a further study, adherence after 1 year was 83% [44] declining to 62–64% after 4–6 years [47, 48]. Recent long-term adherence data demonstrated that 93.3% of the sample (n=172) used the oral appliance for >4 h per night on >4 days per week and 91.3% used the oral appliance for >6 h per night at 5-year follow-up [49]. Patient preference for the continuation of OAT was 96.5% at the 5-year follow-up [49]. However, the study lacked objective adherence data and 48% of initially recruited patients withdrew from the study before the 5-year evaluation which might have confounded the findings of the study. Recently, Vanderveken *et al*. [50] and Johal *et al*. [6] reported on the safety and feasibility, at 3 and 18 months, respectively, of objective measurement techniques with OAT in the same cohort of patients, who demonstrated a range of sleep-disordered breathing, from snoring to OSA [45]. Patient adherence to OAT might be influenced by patient and disease characteristics, type of appliance, *i.e.* custom or ready-made, treatment side-effects and psychosocial factors [51].

Studies exploring the influence of patient and disease characteristics have found no association between objective adherence and anthropometric characteristics, polysomnographic parameters and excessive daytime sleepiness [52]. However, concerning disease characteristics, Nerfeldt and Friberg [53] observed that patients with a greater number of respiratory arousals (arousers) demonstrated higher adherence (85%) than patients with oxygen desaturations (desaturaters). The higher adherence rate in the arousers was attributed to the significant improvement in the Epworth sleepiness scale scores, as compared to the desaturaters. Furthermore, factors such as OAT as the first line of treatment and complete symptom resolution are associated with higher adherence rates [54]. Recent reports also indicate that withdrawal, due to a lack of symptom resolution, is observed more in severe *versus* mild *versus* moderate OSA patients (p=0.008) and obese *versus* nonobese patients (p=0.32) [49]. Interestingly, no studies have been published exploring the association between adherence to OAT and factors such as race and ethnicity-based differences. A low socio-economic index is only considered a barrier to accessing OAT, as its influence on treatment adherence is yet to be explored [55].

In relation to the type of OAT and its influence on patient adherence, studies consistently lean towards a custom-made titratable OAT, as it favours increased patient-reported adherence and patient preference [56–60]. This is in line with the current National Institute of Clinical Excellence guidelines which also recommended custom-made OAT, as lack of retention with the ready-made OAT is the most frequently cited reason for discomfort and nonadherence [5]. Johal *et al*. [59] demonstrated an increased preference and response for custom-made OAT in comparison to ready-made. The authors argued that the “one-size fits all” concept behind ready-made oral appliances might compromise the retention of the appliance since individuals differ morphologically. Likewise, Vanderveken *et al.* [56] reported similar findings with an identical trial. The authors observed a response rate of 60% with custom-made OAT and 30% with ready-made OAT. These response rates are consistent with a three-arm crossover randomised controlled trial (RCT) in which a response rate of 38% and 49% was observed with ready-made and semi-custom-made oral appliances, respectively. Whilst a response rate of 45% was reported in relation to custom-made OAT [58]. Furthermore, patient preference for custom-made OAT is also apparent in the higher number of nights per week and the number of hours per night that the appliance was used [56, 59]. The findings are consistent with a systematic review and meta-analysis comparing custom-made OAT with ready-made OAT [6]. Moreover, as OAT for OSA is entirely dependent on patient behaviour, patient preference cannot be disregarded. However, the above-discussed studies are limited to self-reported use and lack an objective adherence measurement since objective adherence monitors were only employed recently [45, 50].

In relation to the side-effects mentioned above, lack of treatment effects or discomfort and/or pain due to OAT is the most common patient-reported reasons for early discontinuation (<2 years) [48, 61, 62]. Furthermore, such side-effects are observed more with ready-made OAT leading to higher rates of treatment discontinuation, compared to custom-made OAT. Thus, regular follow-up or early intervention of the side-effects is crucial for encouraging patient adherence and for mitigating the risk of early discontinuation [54, 63].

OAT adherence is observed to be significantly associated with psychological and social factors, such as mood and perception of treatment benefits, and bed partner satisfaction levels [64, 65]. Specifically, type D personality, a combination personality type of negative affectivity was found to be negatively attributed to patient adherence [66]. In relation to bed partners, Dieltjens *et al*. [66] found a significant correlation between objective adherence and partner perceived reduction in snoring. Likewise, a holistic improvement in patients and their partner's sleep physiology due to OAT had a positive impact on their emotional and physical relationship [67]. Similarly, Gjerde *et al*. [65] observed that sharing of bedroom in OSA patients can be attributed to increased adherence to OAT. Hence, a partner's perception or their influence is crucial in OSA patients as the therapeutic outcomes of OAT also extend the partners. More recently, patient-tailored therapy in combination with objective adherence monitoring has observed a significant increase in adherence to OAT [68]. Notwithstanding this, evidence in terms of psychological and social factors concerning OAT is highly inconclusive in comparison to CPAP adherence [51].

Furthermore, the abovementioned factors can also aid in predicting the success (baseline apnoea–hypopnoea index (AHI) reduction >50%) rate of OAT and patients may be categorised as responders or nonresponders to treatment. However, successive treatment responders are further divided into AHI<10 and AHI reduction >50% [69]. Studies have consistently observed clinical traits, such as lower age group (<69 years), lower body mass index (BMI), smaller neck circumference and lower AHI in responder groups [49, 69, 70]. Anatomical traits such as a retracted maxilla and mandible are also identified as strong predictors for successful treatment [69–71]. Brown *et al*. [72] observed an *en bloc* (all together) forward movement of the posterior tongue in patients with lower AHI. However, minimal movement of the posterior tongue and increased deformation of the tongue shape was reported in patients with higher AHI when adjusted for BMI. Consequently, responders have demonstrated greater naso- and oropharyngeal anterior tongue movement as compared to nonresponders [73]. Concerning polysomnographic parameters, responders appear to have lower loop gain, whilst low oxygen saturations are seen in nonresponder groups [69, 70, 74, 75]. This is also reflected in patient adherence, as discussed in the above section. Consequently, the above evidence can be applied by sleep clinicians whilst prescribing treatment to patients with OSA. Table 2 summarises detailed evidence concerning factors influencing responders and nonresponders to OAT.

**TABLE 2:** Characteristics of responders and nonresponders to oral appliance therapy

**Characteristics**	**Responders**	**Nonresponders**
**Clinical**	Younger individuals [69, 70] Lower BMI [49, 69, 70] Shorter neck circumference [49, 69, 70] Sex – female [69, 70] Lower prevalence of cardiovascular disease [74]	Older individuals [69, 70] Higher BMI [49, 69, 70] Larger neck circumference [49, 69, 70] Sex – male [69, 70] Increased cardiovascular burden [74]
**Anatomical**	Retracted maxilla or mandible [49, 69, 70] Lower anterior and posterior facial height [69] Shorter distance from the hyoid bone to the third cervical vertebrae [69] Shorter airway length [69] Forward movement of the tongue [72, 73]	Nasal abnormalities [69] Minimal movement of tongue [72, 73]
**Polysomnographic parameters**	Low loop gain [75] Low AHI [75] Higher arousal threshold [75]	Low oxygen desaturations [75]

AHI: apnoea–hypopnoea index; BMI: body mass index.

## Patient preference and treatment effectiveness (*versus* CPAP)

Patient preference is an integral part of patient-centred care as it plays an important role in adherence to and acceptance of treatment and therefore has an impact on health outcomes [76, 77]. There is a move towards gaining a better understanding of patient preferences and values associated with treatment and thus tailor to patient needs and circumstances [76, 78]. Taking patient preference into consideration helps increase patient engagement in the treatment decision-making process and increase their sense of ownership over their treatment [78].

The majority of the trials that compared preference for CPAP with that for OAT found a higher preference for the latter [79]. Five out of the seven crossover trials assessing preference showed higher preference for OAT, one showed slightly more preference for CPAP, while another showed equal preference for both [79, 80].

CPAP has shown to consistently and effectively normalise respiratory parameters and is superior to OAT in doing so [31, 81–84]. It has been better able to reduce AHI compared to OAT, with a mean difference in AHI improvement by six or seven events per hour [31, 83, 85]. Similar trends have been observed with the oxygen desaturation index and minimal oxygen saturation, for which CPAP demonstrated more beneficial effects [81, 83]. Nonetheless, both CPAP and OAT consistently demonstrate similar improvements in symptoms and health-related quality of life measures, which are important components of disease management. Comparable effects on daytime sleepiness and functioning, general physical and mental health, driving simulation assessment and nocturia have been demonstrated by multiple RCTs and meta-analyses [80, 81, 86–89]. Furthermore, the positive effects of CPAP and OAT on daytime sleepiness and functioning continue to be comparable in the long term, as demonstrated by a 10-year follow-up study [90].

The similar positive effect on outcomes that is demonstrated by CPAP and OAT is explained by their comparable effectiveness [79]. It has been hypothesised that the suboptimal efficacy of OAT is compensated for by its superior adherence relative to CPAP, resulting in similar or better overall clinical effectiveness for both treatments [45, 81]. Since the efficacy of a prescribed treatment modality is not synonymous for “being treated” and given that OSA is a chronic condition that requires life-long adherence to therapy, adherence is a major determinant of treatment outcomes. Indices have been developed and utilised to assess the real-world effectiveness of treatment [77]. These indices of effectiveness seem to be better indicators of long-term health benefits and include mean disease alleviation (MDA), sleep-adjusted residual AHI (SARAH index), apnoea burden and effective AHI [50, 79, 91, 92]. They incorporate a measure of efficacy and adherence and, additionally, the latter three incorporate hours of usage relative to total sleep time. Indeed, attempts to compare CPAP and OAT based on these indices of effectiveness have shown comparable or improved MDA and SARAH index for both treatments [45, 50, 91, 93].

OAT has also shown to reduce the intensity and frequency of snoring, as assessed both objectively and subjectively [94–96]. However, there is paucity of research investigating the effect of OAT on fatigue, headaches, anxiety and periodic leg movements. Nevertheless, available data shows a positive effect of OAT on the aforementioned [95–98].

## Impact of treatment on blood pressure and other cardiovascular outcomes

OSA is known to be an independent risk factor for hypertension and cardiovascular disease [99, 100]. OSA causes repeated blood pressure (BP) elevation and tachycardia secondary to sympathetic stimulation [101]. Three meta-analyses [102–104] comparing the effect of CPAP and OAT on BP reduction, showed both modestly improved BP and were comparable in their reduction of both systolic and diastolic BP. Pengo *et al.* [104] in their pooled analysis of RCTs showed a mean BP reduction with CPAP of −2.1 mmHg for systolic and −1.92 mmHg for diastolic BP and a mean BP reduction with OAT of −1.3 mmHg for systolic and −1.1 mmHg for diastolic BP. Furthermore, they identified three subgroups of patients who showed more favourable responses as being those younger than 60 years old, with uncontrolled BP at baseline or with severe oxygen desaturation. The European Respiratory Society guideline found a higher impact of CPAP than of OAT on systolic night-time BP decrease in severe OSA [84]. Albeit seemingly modest, a 2 mmHg reduction in BP has been associated with a 3% reduction in all-cause mortality in the general population [105].

Nocturnal BP nondipping has been associated with incident cardiovascular disease in OSA patients [106], yet only a few studies so far have assessed the effect of OSA treatment on this [103]. A trial comparing CPAP to OAT showed that the frequency of diastolic BP dipping was higher in the OAT group compared to the CPAP group, another trial showed that only 23.5% of nondippers at baseline converted to nocturnal dipping following 1–2 months of OAT [107, 108].

Endothelial dysfunction is a major predictor of late cardiovascular events and has been linked to the severity of OSA [109]. OAT does not seem to have an effect on endothelial dysfunction as indicated by the evidence currently available from RCTs. An RCT that assessed the impact of 2 months of OAT on endothelial dysfunction in severe, nonsleepy OSA patients with no overt cardiovascular disease showed no positive effects [110]. Similarly, a recently published RCT comparing the effect of CPAP and OAT on effectively treated mild OSA patients showed no impact of either treatment on endothelial function or BP, following 1 year of treatment [111]. However, both CPAP and OAT were equally effective in reducing the risk of mortality in patients with severe OSA in an observational study [38, 112]. Additionally, a recently published trial demonstrated reversal of left ventricular hypertrophic remodelling in responders to OAT following 6 months of therapy [113].

Systemic inflammation and metabolic disorders are amongst the mechanisms linking OSA to cardiovascular disease [114]. A trial that prospectively followed up mild to moderate OSA patients for 1 year found improved arterial stiffness, glucose metabolism and insulin resistance with OAT in addition to a reduction in the levels of the inflammatory biomarker fibrinogen [115]. However, an RCT comparing OAT to placebo showed no effect of OAT on circulating inflammatory and metabolic biomarkers in severe OSA patients, with no overt cardiovascular disease following 2 months of OAT [116]. Only a few studies have evaluated the effect of OAT on oxidative stress, one showed a beneficial effect on serum levels of nitric oxide derivatives following 2 months of OAT, while another RCT that assessed 1 month of CPAP and OAT showed no significant changes in most oxidative stress parameters with either therapy [108, 117].

While the potential for positive effects regarding cardiovascular events and mortality cannot be ruled out with CPAP therapy as indicated by observational studies, RCTs (namely the Sleep Apnea Cardiovascular Endpoints trial and the Randomised Intervention with CPAP in Coronary Artery Disease and Sleep Apnea trial) assessing the effects of CPAP on cardiovascular outcomes did not find cardiovascular benefits associated with treatment [118, 119]. This was attributed to the low adherence to therapy (mean adherence of <4 h per night), observed in these trials, yet there was some evidence that there was a reduced risk of adverse events for patients who were adherent to treatment. Other possible explanations for the lack of cardiovascular benefits could be attributed to the short follow up duration in these trials, which was insufficient to express the cardiovascular benefits of therapy [120]. Additionally, the benefits of treatment on cardiovascular event risk may be greater in more symptomatic and more severe disease (severe hypoxemia), which are the groups that were excluded from the RCTs [118, 119, 121]. Therefore, targeting therapy towards specific disease phenotypes may lead to more positive impact on cardiovascular outcomes [104].

Currently, there is a lack of large-scale RCTs comparing the effect of CPAP and OAT on cardiovascular end points and a lack of long-term studies, with greater than 10 years follow-up, assessing the effect of OAT on morbidity and mortality [103, 122]. More research is needed to better understand the role of treatment on both primary and secondary prevention of cardiovascular disease.

## Side-effects of OAT

Side-effects during the first weeks of OAT are common; however, they are generally minor and tend to resolve with time and depend on the OAT design used, being more common with first-generation OAT, due to their inherent inclusion of forward mandibular posturing from the outset. Frequently reported short-term side effects include excessive salivation, mouth dryness, morning-after occlusal changes, difficulty chewing and discomfort in the gums, teeth or jaws [77, 123]. Unlike the short-term ones, the long-term side-effects of OAT are progressive and irreversible. Findings from meta-analyses investigating the nature of these side-effects indicate that the changes that take place in adults are mainly dental, in the form of tooth movements and occlusal changes [124, 125]. A reduction in overjet of 1.9 mm and overbite of 2.3 mm (horizontal and vertical overlap of the incisors, respectively) have been observed following mean treatment duration with OAT of 11 years [126]. The reductions in overjet and overbite occur mainly due to changes in angulation of the upper and lower incisors namely, retroclination of the upper incisors and proclination of the lower incisors [125]. A study that followed up patients for up to 21 years of OAT showed that the retroclination for upper incisors progressed at a relatively constant rate of −0.5 degrees per year, while the rate of proclination of the lower incisors was also progressive up to approximately 19 years [127]. Despite these dental movements, it has been shown that the increase in lower incisor angulation does not induce periodontal disease or bone loss [128].

Other dental changes include mesial tipping of the lower molars (anteriorly repositioned relative to the upper molars), posterior open-bites and anterior cross-bites [124, 125]. Duration of appliance use and the amount of mandibular advancement have been previously linked to the magnitude of side-effects [129, 130]. Despite dental changes being unavoidable regardless of the OAT design, it has been suggested that the features of the appliance may be associated with more pronounced occlusal changes. A study investigating occlusal changes following 2 years of therapy has proposed that OAT in which the adjustment mechanism is located anteriorly were associated with more dental changes compared to those in which the mechanism was located posteriorly [131].

While these long-term side-effects are well tolerated by some patients and mostly go unnoticed, they are not so well tolerated by some other patients and could lead to the discontinuation of treatment. In many instances, even though side-effects may seem substantial, they are outweighed by the benefits gained from OAT. Nonetheless, all patients need to be informed about possible side-effects prior to the initiation of therapy and follow-up of OAT by a qualified dentist is crucial.

## Future considerations in oral appliance therapy treatment in OSA

Oral appliances represent an effective and well-tolerated anatomical type of treatment for selected patients with OSA. In the future, it will be important to improve this selection procedure in order to achieve a more individualised treatment option in relation to other available methods, particularly compared with CPAP with its more stable mechanism of action. The identification of traits that have been associated with success, such as a low loop gain, a high arousal threshold, milder pharyngeal collapsibility, shallower events and tongue base collapse will be simplified by the use of new analysis methods of the respiratory sleep recordings and drug-induced sleep endoscopy [132–135]. It will also become necessary to define treatment success from a wider perspective and use new grading systems that consider a number of variables beyond AHI to identify OAT responders [136, 137]. Improved appliance design including easier and more precise ways to measure and identify the therapeutic mandibular position are also important developments for the future [39, 138, 139]. For the longer-term outcomes, the use of appliances that continuously measure adherence [44] and efficacy would equalise the follow-up regimes with those for CPAP. This will be important to receive a better understanding of the influence of comorbidities and side-effects in terms of bite changes on the longer-term treatment outcome. Furthermore, evidence in relation to the mode of action of OAT is still inconclusive and this is reflected in the current review. Hence, further research is needed to confirm the association between OAT and factors such as upper airway patency and genioglossus muscle activity [19]. Finally, combinations with other types of OSA therapies, such as those that relocate the head or body into more beneficial positions for improved nightly breathing or future therapies that might be available to overcome nonanatomical aetiological traits of OSA, might also increase the usefulness of OAT in the treatment of various groups of OSA patients [140–142].
